# Physical Activity and Sedentary Behaviors of Young Children: Trends from 2009 to 2018

**DOI:** 10.3390/ijerph17051645

**Published:** 2020-03-03

**Authors:** Fotini Venetsanou, Kyriaki Emmanouilidou, Olga Kouli, Evangelos Bebetsos, Nikolaos Comoutos, Antonis Kambas

**Affiliations:** 1 School of Physical Education and Sport Science, National and Kapodistrian University of Athens, 17237 Athens, Greece; fvenetsanou@phed.uoa.gr; 2 School of Physical Education and Sport Science, Democritus University of Thrace, 69100 Komotini, Greece; kemmanou@phyed.duth.gr (K.E.); okouli@phyed.duth.gr (O.K.); empempet@phyed.duth.gr (E.B.); 3 Department of Physical Education and Sport Science, University of Thessaly, 42100 Trikala, Greece; nzourba@uth.gr

**Keywords:** health behaviors, screen time, financial crisis, family affluence, recession, pedometer, FAS, preschool

## Abstract

Over the last decade, the lives of children in several countries, including Greece, have been affected by recession. The aim of the present study was (a) to examine time trends in physical activity (PA) and screen time (ST) of Greek preschool children, together with their family affluence (FA), from 2009 until 2018, and to explore the associations among them; and (b) to investigate the connections of parental educational level and children’s BMIs to their achieving ST (<1 h/day) and PA (11,500 steps/day) guidelines. A total of 652 children from four cross-sectional cohorts participated. PA was recorded with Omron HJ-720IT-E2 pedometers, whereas ST, family affluence (FA) and parental educational level were reported by participants’ parents. The results of the one-way ANOVAs that were computed revealed statistically significant differences among cohorts, albeit of no practical importance, in PA, ST and FA. According to the regressions calculated, neither BMI nor the educational level was related to membership in ST and PA guidelines groups. ST was a significant predictor of children’s PA in all week periods (school-time, leisure-time, weekend), whereas FA was not such a strong predictor. Multilevel interventions aiming at both ST and PA seem to be imperative for the benefit of young children’s health.

## 1. Introduction

Preschool years are vital for the configuration of behaviors that are closely tied to health, such as those of physical activity (PA) [1] and screen use [2]. To begin with, PA is thought to be a cornerstone for several health aspects [3,4], protecting against obesity and non-communicable diseases [5]. On the contrary, excessive screen time (ST) not only negatively associates with preschoolers’ PA [6,7], but also harms their metabolic health [8], and their motor [9], cognitive [9,10] and psychosocial development [11].

The importance of the above behaviors in young children’s lives has led several scientific associations to the establishment of PA and ST guidelines aiming at safeguarding public health. Regarding PA, it is recommended that preschool children should accumulate at least 180 min of PA [12,13] or 11,500 steps [14] every day. Conversely, ST for 2–5-year-old children should be limited to one hour per day [15,16]. Nevertheless, despite the aforementioned published guidelines, today’s young children have been found to be both physically inactive [7,17,18,19] and heavily exposed to screens [7,20,21,22,23].

Among the factors that are associated with young children’s health behaviors, parents seem to be among the most crucial, being not only “gatekeepers” but also role models for their offspring [24]. As far as the role of parents’ socio-economic status (SES) is concerned, research findings are conflicting, with some studies reporting that children with higher parental SES present higher PA levels [25] and lower ST [26,27], whereas a number of studies have found no association between SES and either PA [28] or ST [29,30]. Similarly unclear are the findings concerning the role of family affluence (FA), as an indicator of SES, on children’s ST [15] or PA [31], although it would be expected to be a predictor of children’s PA, since nowadays, organized PA is mostly paid for [32]. Moreover, available facilities triggering PA, such as playgrounds and parks in the neighborhood, have been found to enhance parental support to their offspring’s PA [33,34]. Apart from family, preschool settings can be important contributors to PA [35] and ST [36], since they host young children for a large period of their day.

During the last decade, children’s lives have been affected by the recession which started in the United States and quickly spread around the world [37]. In Europe, rapid increases in the unemployment rate and austerity measures introduced by governments have negatively affected children and their families’ well-being [38], to varying degrees among countries, however, with the south of Europe, the Baltic States and Croatia presenting the most severe child poverty [37]. One of the most heavily affected countries is Greece, having faced a deep and persistent recession since 2009 [39], with a sharp rise in unemployment that remains at a very high level (in 2018, the long term unemployment in Greece was 13.6% with the equivalent rate in the EU being 2.9%) [40]. Children’s health-related behaviors are expected to have been affected by the deterioration in their quality of life, especially in the countries that have been hard hit. Nevertheless, until now studies have focused on the effects of financial crisis only on adults’ PA [41,42]. Although children are among the most vulnerable populations [38], there is no evidence regarding them. Taking into account both the negative impact of the recession on children’s quality of life and the importance of preschool age for the establishment of PA and screen use behaviors, a study investigating PA and ST of preschoolers in a country having experienced the consequences of years of financial crisis would provide valuable evidence. On this basis, the aim of the present study was (a) to examine time trends in PA and ST of Greek preschool children, along with their FA, from the beginning of the recession (2009) until recently (2018), and to explore the associations among them; and (b) to investigate the connections of parental educational level and children’s body mass index (BMI) to their achieving ST and PA recommendations.

## 2. Materials and Methods

### 2.1. Participants

A total of 652 children from four cross-sectional cohorts (2009–2018) attending childcare centers in Northern Greece participated in the study. In 2009, 48 public childcare centers were selected via stratified sampling, using area (rural, urban) as stratification variable. After contacting their directors, 24 childcare centers agreed to participate. Data were gathered from children hosted in these 24 childcare centers, in 2009 (*n* = 182 (72 boys, 110 girls)); 2012 (*n* = 161 (62 boys, 99 girls)); 2015 (*n* = 165 (65 boys, 100 girls)) and 2018 (*n* = 144 (61 boys, 83 girls)). Informed consent was obtained from both parents/legal guardians and children’s teachers who volunteered to participate (corresponding rate ranged from 48% (2018) to 65% (2009)). The study was approved by the Ethics Committee of the Master’s degree of the Department of Physical Education and Sport Science of the Democritus University of Thrace (11/2009, 02/2012, 7/2015, 14/2018).

### 2.2. Measurements

#### 2.2.1. Physical Activity

Children’s PA was measured with Omron Walking style pro HJ-720IT-E2 pedometers (Omron Corp, Kyoto, Japan) that have been found to accurately record young children’s ambulatory activity [43]. With this type of pedometer, data can be downloaded hour-by-hour, a feature that had a two-fold contribution to the study. Firstly, pedometer removal for more than one hour could be detected. In such cases, participants were excluded from the sample [44]. Secondly, it was possible to classify children’s weekday ambulatory activity to PA during school-time and leisure-time (time after school). For the purposes of this study, children’s PA was recorded for the following time periods: (a) school-time, (b) leisure-time (at least three weekdays data were necessary for the calculation of both (a) and (b) average step counts), (c) weekends (data gathered on at least one weekend day were necessary for average weekend step counts) and (d) weekly (average step counts for at least four days).

#### 2.2.2. Socio-Economic Characteristics

Parents were asked to provide information about their educational levels via a three-item list (primary education (6 years); secondary education (9–12 years); higher education (≥15 years)). Besides the parental educational level, the Family Affluence Scale (FAS) [45,46] was used to assess SES, since, based on family consumption, it appears to be more ecologically valid than parental income [47,48]. The FAS has been validated and used in several countries, including Greece [48]. It consists of four items: (a) number of cars in the family, (b) child’s personal bedroom, (c) number of personal computers and (d) number of family vacations during the previous 12 months. Responses in FAS items are categorized from 0 to 3 (0–2 for (a); 0–1 for (b); 0–3 for (c); 0–3 for (d)), and an FA composite score is computed, according to which, FA is characterized as low (0–2), medium (4 and 5) or high (6–9).

#### 2.2.3. Screen Time

Parents were asked about their children’s ST via the following question: “On a typical week day, how long time does your child usually spend on TV (watching television or videos) and/or a computer/other smart devices, including playing computer games and using the Internet?” (not including time spent at school). Parents’ responses were given in hours and minutes. This question has been used for children’s ST assessment in several studies [7,49,50,51] and presented acceptable test-retest reliability (intraclass correlation = 0.68, 95% CI = 0.52–0.83) [49].

#### 2.2.4. Anthropometry

Children’s standing heights were measured to the nearest 0.5 cm (Stadiometer 208, Seca, UK), whereas their body masses were measured to the nearest 0.1 kg, using a mechanical scale (Beam Balance 710, Seca, UK). BMI was calculated, utilizing the formula body mass/height^2^ (Kg/m^2^).

### 2.3. Procedure

Data collection was conducted every three years from 2009 until 2018 during September and October. At first, a briefing meeting with children’s teachers and parents took place at each of the 24 childcare centers that agreed to participate in the study, on the first fifteen days of each September with measurements. Parents who volunteered to participate were invited to a second meeting that took place one week later. During that meeting, parents anonymously completed the structured questionnaires regarding their SES and their offspring’s ST and age. Furthermore, they were provided with detailed instructions about pedometer placement and operation. Participants should wear their pedometer on their right hip all day long except the hours of sleeping and water activities. Children’s PA was assessed for 10 days. Among them, the first three days were used for children’s familiarization with the pedometer (those data were not used for statistical analysis). Pedometers were collected by the researchers on the eleventh day. Only data of participants having worn their pedometers for at least four valid days (≥7 h/day) were used in the statistical analyses. Children’s standing heights and body masses were measured in a quiet room at childcare centers, each child individually, by a trained assessor of the same sex. During anthropometric measurements the examinee was barefooted and wore his/her underclothes.

### 2.4. Data Analysis

Descriptive data of children and their parents are presented as means (± standard deviations) or percentages per cohort. Since cluster analysis did not reveal any indicator for clustering by childcare center in the variables of interest in any of the cohorts, single-level analyses were computed. At a preliminary level, independent sample t-tests were utilized on each cohort data to check potential gender differences on PA. Since no practical significant differences were detected (*η*^2^ = 0.03), data of boys and girls were merged. In the next step, one-way analyses of variance were computed on children’s step counts (school steps; leisure-time steps; weekend steps), FAS and ST to check for potential differences between cohorts (2009 vs. 2012 vs. 2015 vs. 2018). For post-hoc comparisons, Bonferroni test was applied. Moreover, the effect size was also computed, using eta squared values. To estimate effect size, Cohen’s criteria were followed (only *η*^2^ ≥ 0.14 are considered to show a practically important association; [52]). Then, correlates of children’s PA and ST were investigated. Firstly, logistic regressions using the child’s BMI and paternal and maternal educational level as predictors were computed to predict meeting PA and ST guidelines. Secondly, multiple linear regressions were computed to predict children’s school steps, leisure steps and weekend steps based on their FAS and ST values. For all the statistical analyses the alpha level was set at 0.05. All statistical analyses were performed with the SPSS v21.0 software (IBM SPSS, Inc., Chicago, IL, USA).

## 3. Results

In Table 1, the means and standard deviations of (a) parents’ educational levels; and (b) children’s age, PA (school-time, leisure-time, weekend, weekly), ST and FA values, and frequencies of FA categories and children meeting guidelines for ST (<1h/day; [15,16]) and PA (11,500 steps/day; [14]) are presented.

**Figure 1:**
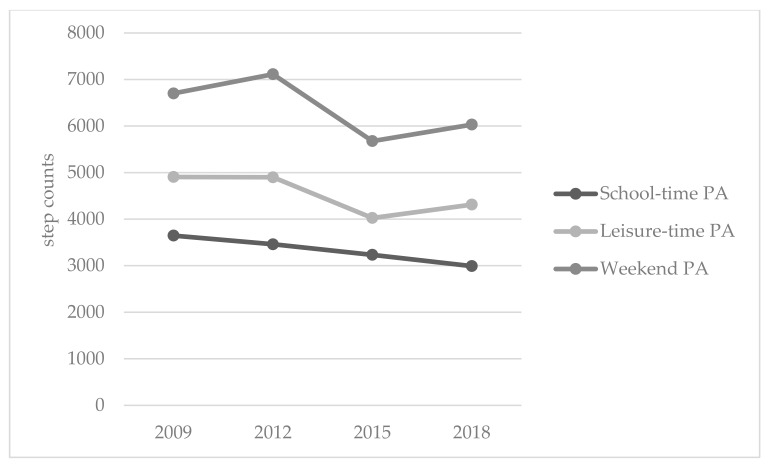
Mean step counts during school-time, leisure-time and weekends among Greek preschool children between 2009 and 2018.

**Figure 2:**
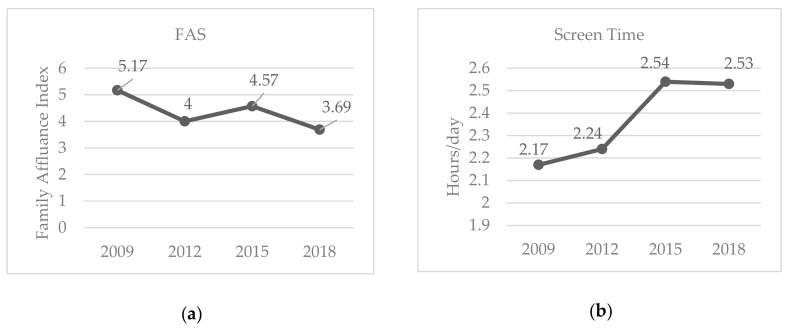
Mean FAS and ST scores among Greek preschool children between 2009 and 2018: (**a**) mean FAS scores for Greek preschool children between 2009 and 2018; (**b**) mean ST scores among Greek preschool children between 2009 and 2018.

The ANOVAs revealed statistically significant differences among cohorts, although with low to moderate eta squared values, in leisure-time PA (F = 9.49, *p* < 0.001, *η*^2^ = 0.07), school-time PA (F = 4.00, *p* = 0.008, *η*^2^ = 0.031) and weekend PA (F = 4.75, *p* = 0.003, *η*^2^ = 0.036) (Table 1; Figure 1). Regarding FAS, statistically significant differences were found among cohorts, although with moderate eta squared value (F = 29.33, *p* < 0.001, η^2^ = 0.12) (Figure 2). Finally, according to the results of the ANOVA computed on children’s ST, there were statistically significant differences among cohorts (F = 9.66, *p* < 0.001, η^2^ = 0.053) (Figure 2).

The logistic regression results indicated that children’s BMIs and parents’ educational levels were unrelated to children’s membership in ST and PA guideline groups (*p* > 0.05 for all independent variables). In the multiple linear regression computed on children’s school steps, a significant regression equation was found (F_2,485_ = 118.62, *p* < 0.001), with an R^2^ of 0.361 (predicted school steps = 5525.73 + 131.21*FAS − 1203.41*ST). Both FAS and ST were significant predictors (*t* = 3.86, *p* < 0.001 and t = −15.25, *p* < 0.001, for FAS and ST respectively). Regarding children’s leisure-time steps, a significant regression equation was found (F_2,485_ = 238.19, *p* < 0.001), with an R^2^ of 0.496 (predicted leisure-time steps = 7846.07 + 39.94*FAS − 1483.83*ST). However, only ST was a significant predictor (*t* = −21.83, *p* < 0.001), whereas FAS was not (*t* = 1.41, *p* = 0.16). As far as weekend steps are concerned, a significant regression equation was found (F_2,485_ = 278.58, *p* < 0.001), with an R^2^ of 0.545 (predicted weekend steps= 14518.96 − 142.62*FAS − 3189.00*ST). Both FAS and ST were significant predictors (*t* = −2.41, *p* = 0.016 and *t* =-23.17, *p* < 0.001, for FAS and ST respectively).

## 4. Discussion

It is well known that the recession has had negative consequences on children’s well-being [38]; thus, their health behaviors are expected to have been also affected. Nevertheless, to our knowledge, to date there has been no research evidence regarding young children’s PA and ST—their correlation—in the years of the recession. Taking into account that preschool age constitutes a key-period for the establishment of health behaviors [1], the aim of this study was to examine the associations among PA, ST, BMI, FA and parental educational level in Greek preschool children, between 2009 and 2018, a period in which Greece faced a deep financial crisis.

A key-finding of this study was the very low percentage of Greek children who met the PA limit (11,500 steps/day [14]) or the ST limit (≤1 h/day [15,16]), both of which are important for their health. Starting with PA, children in every cohort presented low step counts at every phase of the week, confirming previous Greek studies that reported poor PA levels during preschool-hours (3280 ± 1014 steps/day [18]; 2564 ± 921 steps/day [53]), leisure-time (5911 ± 1403 steps/day [18]) and weekends (7913 ± 2181 steps/day [18]). Although there was a decline from cohort to cohort, with some small fluctuations in weekend and leisure-time PA, what should be mentioned is the low ambulatory activity presented in every cohort that led to a very low percentage of children (3.8%–6%) meeting the steps/day guideline [14].

As far as screen use is concerned, a high prevalence of excessive ST was revealed, with children of the four cohorts being, on average, exposed to screens more than 2 h/day. Taking into account both that in the present study ST was reported by children’s parents (thus, no information about school ST was gathered), and that children seem to accumulate approximately one h/day of ST during school hours [54], it can be assumed that the total ST of our participants was at even higher levels. In previous Greek studies [7,55], 32%–63.3% of children were found to exceed the 2-h ST limit [56]. In this study, where the 1-h ST limit [15,16] was used, a higher percentage of children (80%–90%) were found not to meet it. Moreover, a fractional percentage of children met the recommendations for both PA and ST.

The above findings can be partially explained under the prism of the prolonged financial crisis that Greece has been facing since 2009 [39,57]. During these years, a sharp rise in unemployment rates [40,58] and income loss for the vast majority of households [59], the highest in EU and OECD [60], took place and were accompanied by severe cuts in public expenditure, painful across-the-board salary cuts and serious tax increases [61]. The aforementioned vitally harmed several aspects of children’s lives. To begin with, at the governmental (both national and local) level, although it is often supported that facilities and programs promoting PA and quality of life are worth the money spent on them, they are among the first to face cuts in a financial resource scarcity [62]. Focusing on preschool education, due to public expenditure cuts, preschool settings in Greece have been stripped of equipment (both stable and portable) [53] that could help children to be more active, whereas PE or organized PA programs are not offered [63].

At a family level, there has been inevitable affluence deterioration that was reflected in our results with statistical and almost practical significant differences in FAS scores among cohorts. Greek families have been reported to be facing several problems, with the biggest ones being those of paying utility bills, coping with unexpected expenses and dealing with a lack of leisure time [64]. In an attempt to confront the difficulties their household faces, parents seem to develop several strategies, among which are cutting children’s extra activities, such as participation in sports [38], viewing their costs as barriers, regardless of their FA [65]. That explains our inconsistent findings regarding the association between FAS and PA (it is positively associated with children’s PA during school-time, but negatively associated with weekend PA (though, with a small contribution to the predicted step counts), whereas it did not predict leisure-time PA).

Apart from the above, the parents’ important role for their young children’s health behaviors [24] should not be overlooked. In periods of unemployment, job insecurity and general anxiety, parents’ leisure time and personal care has been found to diminish [66], resulting in increased prevalence of unhealthy behaviors, such as drinking, smoking and physical inactivity [55]. Moreover, due to the unstable working environment, parents tend to work at multiple jobs or increase job searching [66,67]. For that reason, they frequently leave the care of their younger offspring to older siblings or other family members [67], and when they return home from work they are often too tired to play or to engage in PA with their preschool aged children [68].

All the aforesaid negative conditions have significantly contributed to our participants’ physical inactivity. However, Greek children seemed to be physically inactive before the recession. Although relative research evidence is very limited, it reveals that even in 2005, Greek preschool children presented low PA (8871 ± 2948 steps/day; 81.7% not meeting the 11,500 steps/day guideline) and excessive ST (30.1% exceeded two hours of ST per day) [7], a finding that was also noticed in 2004 (32%; [55]). It appears that Greek children’s health behaviors, having already been at worrying levels, deteriorated during the recession.

A second key-finding of this study that adds another piece to the puzzle of the chronic physical inactivity of Greek children is the strong association between ST and PA. Both in the present study and in previous ones with preschoolers [6,7], screen overuse was connected to low PA. It is well known that excessive ST is associated with several health risks not only in the short but also in the long term [69], and reduced PA is only one of them. Moreover, although in the present study, children’s BMIs were proven to be insignificant for their PA or ST, the risk of being overweight due to early excessive ST that can persist later in life is underlined [8].

Unfortunately, numerous studies report that today’s children worldwide are exposed to screens two or more hours daily [26,70,71,72,73], whereas exceeding 4 h/day [74] or even 5 h/day [23] is no longer surprising. It appears that screen overuse is a global phenomenon that is largely irrespective of the recession, since high ST levels are reported in several countries in which child poverty was not increased [75], including Canada [70] and Australia [71]. Nowadays, exposure to screens starts as early as infancy [76] (in high-income countries, children younger than five years exceed one hour/day of ST [77]). This can only to be expected due to the extensive availability of electronic devices—smartphones, tablets, etc. [78].

Parents are the first to be held responsible for its restriction. In the previous decade, parents were found to ignore ST limits [79] and believed that their children benefitted from media use [80]. However, it seems that they still hold the same beliefs [8]. Perhaps that is why they often use screens as babysitters for their preschool aged children both at home, so they can perform household tasks or rest [67], and also when eating out [81]. At this point, it is interesting to note that in the present study and in previous ones [29,30], no relation was found between parents’ educational levels and children’s meeting ST or PA guidelines. Taking into account the multiple negative consequences of excessive ST, interventions aiming at raising parents’ awareness about this phenomenon seem imperative. It is necessary for parents to understand that efficient screen use parenting results in less ST [82]. Additionally, it is important for parents to understand that their own screen use behaviors influence those of their children [83].

Furthermore, parents need to be aware of their important role as PA models. This study and several others [20,84] revealed that at weekends young children present low PA levels, with the vast majority (97%) spending their time on sedentary behaviors [20,85]. Taking into consideration that, at the weekends, young children share their leisure-time with their parents, this finding shows the negative influence of parents on their offspring’s PA and thus calls for action.

However, it is well known that with regard to PA, parents should not be the only target of interventions. Agreeing with several authors [7,86,87], we strongly believe that PA policies at childcare centers could be an effective means for children’s PA enhancement. Although in some studies a high PA is reported [20], in agreement with our results, a recent literature review [88] showed that the majority (>50%) of the preschool day is sedentary. In Greece, as in several other countries (e.g., Canada [86] and New Zealand [89]), there are no established policies regarding PA and sedentary time at preschool education. What is more, at Greek childcare centers only some general pedagogical guidelines are provided, since there is no national curriculum [90]. That undermines the quality of early education, jeopardizing not only children’s ST and PA but also their optimal development [60]. In a recent Greek study [53] it was revealed that preschool teachers do not implement any PA policies. This is unsurprising, since preschool teachers in Greece have been neither educated [63] nor reeducated [53] in the fields of physical education or PA. The need for preschool staff training has been noticed by several authors [63,86] and is of primary importance if childcare centers are to become “active schools.”

This study has some limitations that should be taken into consideration when interpreting its findings. To begin with, it is well known that pedometers, although an objective means for PA recording, do not provide useful information about the type and intensity of PA. Moreover, as was reported earlier in this manuscript, data regarding children’s ST were gathered only by parents who provided information about average ST of their children; thus, there is no information about ST during school hours or discrimination between weekdays and weekend ST. Last, the study design of this research (comparison of data from four cross-sectional cohorts) does not allow for testing causal relationships. Nonetheless, despite its limitations, this study is the first to examine young children’s health behaviors, such as those of PA and ST, in a country experiencing a prolonged financial crisis that affected several aspects of its citizens’ lives. Furthermore, the associations of children’s PA and ST with sociodemographic factors were investigated, shedding light on a very important issue.

## 5. Conclusions

This study demonstrated that from 2009 until 2018, when Greece faced a deep financial crisis, young children presented low PA and high ST levels, with a very high percentage not succeeding in meeting ST and PA limits that are vital for their health. However, the above findings can be only partially interpreted by the consequences of the financial crisis on families’ lives. Greek children have been reported to present worrying PA and ST levels before 2009, which deteriorated due to the recession. Taking into account that ST was a significant predictor of children’s PA, whereas FA provided inconsistent findings and parents’ educational level did not associate with children’s PA and ST, it seems that the Greek family has not developed a “screen and PA culture” as yet. Moreover, relevant policies have not been implemented either on local or national level.

It is more than obvious that effective interventions aiming at reducing ST and enhancing PA at an early age are imperative for the benefit of children’s health. In order to further this aim, national PA planning would be beneficial. In view of the poor economy of Greece, investments in parks, bike paths and sport programs or payment for PA in health care, etc., all of which have been proven to enhance PA levels [31], seem difficult to implement. Nevertheless, the cooperation of the state with the university to organize campaigns targeting parents, create a PA-centered curriculum at childcare centers, train preschool teachers on integrating PA into school day and educate people towards an active life style, so as to cultivate PA-friendly cultural values, could be both feasible and fruitful.

## Figures and Tables

**Table 1:** Descriptive statistics for participants by cohort.

	2009 (*n* = 182)	2012 (*n* = 161)	2015 (*n* = 165)	2018 (*n* = 144)	Total (*n* = 652)
Child’s age (months)	52.58 ± 3.55	52.50 ± 3.51	53.02 ± 3.58	52.79 ± 3.57	52.72 ± 3.55
Child’s BMI (Kg/m^2^)	16.71 ± 1.69	16.71 ± 1.70	17.05 ± 1.98	16.61 ± 1.62	16.78 ± 1.76
Maternal educational level (%)					
Primary education	0/%	0/%	0/%	0/%	0/%
Secondary education	50.6%	56.0%	48.4%	49.3%	51.1%
Higher Education	49.4%	44.0%	51.6%	50.7%	48.9%
Paternal educational level (%)					
Primary education	2.3%	3.1%	4.3%	0.7%	2.7%
Secondary education	55.7%	59.7%	49.7%	54.2%	54.9%
Higher Education	42.0%	37.1%	46.0%	45.1%	42.5%
FAS	5.17 ± 1.27 ^a^	4.00 ± 1.65 ^b,d^	4.57 ± 1.73 ^a^	3.69 ± 1.54 ^b,d^	4.40 ± 1.65
Weekly PA (step counts)	8032 ± 2026	7816 ± 2087	6708 ± 2739	6943 ± 2729	7309 ± 2512
School-time PA (step counts)	3646 ± 1372 ^e^	3459 ± 1175	3233 ± 1590	299 1 ± 1433 ^b^	3309 ± 1442
Leisure-time PA (step counts)	4906 ± 1300 ^d,e^	4899 ± 1321 ^d^	4026 ± 1531 ^b,c^	4312 ± 1466 ^b^	4470 ± 1471
Weekend PA (step counts)	6700 ± 2914 ^d^	7112 ± 2802 ^d^	5676 ± 3321 ^b,c^	6031 ± 3412	6419 ± 3185
ST	2.17 ± 0.73 ^d,e^	2.24 ± 0.71 ^d,e^	2.54 ± 0.67 ^b,c^	2.53 ± 0.67 ^b,c^	2.40 ± 0.71
Meeting PA guideline (%)	3.8%	3.9%	4.2%	6.0%	4.5%
Meeting ST guideline (%)	19.1%	15.5%	9.8%	9.7%	12.9%
Meeting both PA and ST guidelines (%)	3.3%	2.5%	4.2%	2.8%	3.2%

PA pedometer determined physical activity, ST screen time, FAS family affluence scale index; ^a^ significantly different from the other cohorts; ^b^ significantly different from 2009 cohort; ^c^ significantly different from 2012 cohort; ^d^ significantly different from 2015 cohort; ^e^ significantly different from 2018 cohort.

## References

[1] Goldfield G.S., Harvey A., Grattan K., Adamo K.B. (2012). Physical activity promotion in the preschool years: A critical period to intervene. Int. J. Environ. Res. Public Health.

[2] American Academy of Pediatrics, Council on Communications and Media (2016). Media and young minds. Pediatrics.

[3] Timmons B.W., LeBlanc A.G., Carson V., Connor Gorber S., Dillman C., Janssen I., Kho M.E., Spence J.C., Stearns J.A., Tremblay M.S. (2012). Systematic review of physical activity and health in the early years (aged 0–4 years). Appl. Physiol. Nutr. Metab..

[4] Venetsanou F., Kambas A., Giannakidou D. (2015). Organized physical activity and health in preschool age: A review. Cent. Eur. J. Public Health.

[5] Finger J.D., Varnaccia G., Borrmann A., Lange C., Mensink G. (2018). Physical activity among children and adolescents in Germany. Results of the cross-sectional KiGGS Wave 2 study and trends. J. Health Monit..

[6] Wenhold H., Harrison K. (2019). Familial correlates of US preschooler physical activity. J. Child. Media.

[7] Venetsanou F., Kambas A., Gourgoulis V., Yannakoulia M. (2019). Physical activity in pre-school children: Trends over time and associations with body mass index and screen time. Ann. Hum. Biol..

[8] Kostyrka-Allchorne K., Cooper N.R., Simpson A. (2017). Touchscreen generation: children’s current media use, parental supervision methods and attitudes towards contemporary media. Acta Paediatr..

[9] Lin L.Y., Cherng R.J., Chen Y.J., Chen Y.J., Yang H.M. (2015). Effects of television exposure on developmental skills among young children. Infant Behav. Dev..

[10] Collet M., Gagnière B., Rousseau C., Chapron A., Fiquet L., Certain C. (2019). Case–control study found that primary language disorders were associated with screen exposure. Acta Paediatr..

[11] Carson V., Lee E.-Y., Hesketh K.D., Hunter S., Kuzik N., Predy M., Rhodes R.E., Rinaldi C.M., Spence J.C., Hinkley T. (2019). Physical activity and sedentary behavior across three time-points and associations with social skills in early childhood. BMC Public Health.

[12] Department of Health, Physical Activity (2011). Health Improvement and Protection. Start active, stay active. A Report on Physical Activity for Health from the Four Home Countries’ Chief Medical Officers.

[13] Institute of Medicine of the National Academies (2011). Early Childhood Obesity Prevention Policies.

[14] De Craemer M., De Decker E., De Bourdeaudhuij I., Verloigne M., Manios Y., Cardon G. (2015). The translation of preschoolers’ physical activity guidelines into a daily step count target. J. Sports Sci..

[15] Canadian Paediatric Society (2017). Screen time and young children: Promoting health and development in a digital world. Paediatr. Child Health.

[16] World Health Organization (WHO) (2019). Guidelines on Physical Activity, Sedentary Behaviour and Sleep for Children under 5 Years of Age.

[17] Hallal P.C., Andersen L.B., Bull F.C., Guthold R., Haskell W., Ekelund U. (2012). Global physical activity levels: Surveillance progress, pitfalls, and prospects. Lancet.

[18] Kambas A., Venetsanou F., Avloniti A., Giannakidou D.M., Gourgoulis V., Draganidis D., Chatzinikolaou A., Fatouros I., Michalopoulou M. (2015). Pedometer determined physical activity and obesity prevalence of Greek children aged 4–6 years. Ann. Hum. Biol..

[19] Voukia C., Voutsina I., Venetsanou F., Kambas A. (2018). Child and parental physical activity: Is there an association with young children activity?. Cent. Eur. J. Public Health.

[20] Berglind D., Tynelius P. (2017). Objectively measured physical activity patterns, sedentary time and parent-reported screen-time across the day in four-year-old Swedish children. BMC Public Health.

[21] Hinkley T., Brown H., Carson V., Teychenne M. (2018). Cross sectional associations of screen time and outdoor play with social skills in preschool children. PLoS ONE.

[22] Sigmundová D., Badura P., Sigmund E., Bucksch J. (2018). Weekday–weekend variations in mother-/father–child physical activity and screen time relationship: A cross-sectional study in a random sample of Czech families with 5- to 12-year-old children. Eur. J. Sport Sci..

[23] Webster E.K., Martin C.K., Staiano A.E. (2019). Fundamental motor skills, screen-time, and physical activity in preschoolers. J. Sport Health Sci..

[24] Yao C.A., Rhodes R.E. (2015). Parental correlates in child and adolescent physical activity: A meta-analysis. Int. J. Behav. Nutr. Phys. Act..

[25] Mutz M., Albrecht P. (2017). Parents’ Social Status and Children’s Daily Physical Activity: The Role of Familial Socialization and Support. J. Child Fam. Stud..

[26] Chiu Y.-C., Li Y.-F., Wu W.-C., Chiang T-L. (2017). The amount of television that infants and their parents watched influenced children’s viewing habits when they got older. Acta Paediatr..

[27] Loprinzi P.D., Schary D.P., Cardinal B.J. (2013). Adherence to active play and electronic media guidelines in preschool children: Gender and parental education considerations. Matern. Child Health J..

[28] O’Donoghue G., Kennedy A., Puggina A., Aleksovska K., Buck C., Burns C., Cardon G., Carlin A., Ciarapica D., Colotto M. (2018). Socioeconomic determinants of physical activity across the life course: A “DEterminants of DIet and Physical ACtivity” (DEDIPAC) umbrella literature review. PLoS ONE.

[29] Bergqvist L. (2016). Objectively Measured Physical Activity in Three-Year-Old Children—Associations with BMI, Gender and Parental Socioeconomic Status. Master’S Thesis.

[30] Carson V., Kuzik N. (2017). Demographic correlates of screen time and objectively measured sedentary time and physical activity among toddlers: A cross-sectional study. BMC Public Health.

[31] Bauman A.E., Reis R.S., Sallis J.F., Wells J.C., Loos R.J., Martin B.W., Lancet Physical Activity Series Working Group (2012). Correlates of physical activity: Why are some people physically active and others not?. Lancet.

[32] World Health Organisation (WHO) (2016). Growing up unequal: Gender and socioeconomic differences in young people’s health and well-being (HBSC) study: International report from the 2013/2014 survey. Health Policy Child. Adolesc..

[33] Lu C., Huang G., Corpeleijn E. (2019). Environmental correlates of sedentary time and physical activity in preschool children living in a relatively rural setting in the Netherlands: A crosssectional analysis of the GECKO Drenthe cohort. BMJ Open.

[34] Rossi C.E., Patrícia de Fragas H., Corrêa E.N., das Neves J., de Vasconcelos F.A.G. (2018). Association between food, physical activity, and social assistance environments and the body mass index of school children from different socioeconomic strata. J. Public Health (Oxf.).

[35] Van Cauwenberghe E., De Craemer M., De Decker E., De Bourdeaudhuij I., Cardon G. (2013). The impact of a teacher-led structured physical activity session on preschoolers’ sedentary and physical activity levels. J. Sci. Med. Sport.

[36] Staiano A.E., Webster E.K., Allen A.T., Jarrell A.R., Martin C.K. (2018). Screen-time policies and practices in early care and education centers in relationship to child physical activity. Child. Obes..

[37] UNESCO (2014). Early Childhood Care and Education: Addressing Quality in Formal Pre-Primary Learning Environments.

[38] Ruxton S. (2012). How the economic and financial crisis is affecting children & young people in Europe. Eurochild Report Prepared under European Community Programme for Employment and Social Solidarity.

[39] Papadakis N., Amanaki E., Drakaki M., Saridaki S. (2020). Employment/unemployment, education and poverty in the Greek Youth, within the EU context. Int. J. Educ. Res..

[40] Eurostat Long-Term Unemployment Rate by Sex (Tesem130). https://ec.europa.eu/eurostat/databrowser/view/sdg_08_40/default/table?lang=en.

[41] Filippidis F.T., Gerovasili V., Millett C., Tountas Y. (2017). Medium-term impact of the economic crisis on mortality, health-related behaviours and access to healthcare in Greece. Sci. Rep..

[42] Macassa G., Ahmadi N., Alfredsson J., Barros H., Soares J., Stankunas M. (2016). Employment status and differences in physical activity behavior during times of economic hardship: Results of a population-based study. Int. J. Med. Sci. Public Health.

[43] Venetsanou F., Kambas A., Giannakidou D.M., Avloniti A., Draganidis D., Chatzinikolaou A., Fatouros I., Michalopoulou M. (2015). The validity of two Omron pedometers in preschool children under different conditions. Sylwan.

[44] Tudor-Locke C., Pangrazi R.P., Corbin C.B., Rutherford W.J., Vincent S.D., Raustorp A., Tomson L.M., Cuddihy T.F. (2004). BMI-referenced standards for recommended pedometer-determined steps/day in children. Prev. Med..

[45] Currie C., Molcho M., Boyce W., Holstein B., Torsheim T., Richter M. (2008). Researching health inequalities in adolescents: The development of the Health Behaviour in School-Aged Children (HBSC) family affluence scale. Soc. Sci. Med..

[46] Boyce W., Torsheim T., Currie C., Zambon A. (2006). The family affluence scale as a measure of national wealth: Validation of an adolescent self-report measure. Soc. Indic. Res..

[47] Kim H.N., Kim J.H., Kim S.Y., Kim J.B. (2017). Associations of community water fluoridation with caries prevalence and oral health inequality in children. Int. J. Environ. Res. Public Health.

[48] Yannakoulia M., Lykou A., Kastorini C., Saranti Papasaranti E., Petralias A., Veloudaki A., Linos A. (2016). Socio-economic and lifestyle parameters associated with diet quality of children and adolescents using classification and regression tree analysis: The DIATROFI study. Public Health Nutr..

[49] Hinkley T., Salmon J.O., Okely A.D., Crawford D., Hesketh K. (2012). Preschoolers’ Physical Activity, Screen Time, and Compliance with Recommendations. Med. Sci. Sports Exerc..

[50] Frate N., Jenull B., Birnbacher R. (2019). Like Father, Like Son. Physical Activity, Dietary Intake, and Media Consumption in Pre-School-Aged Children. Int. J. Environ. Res. Public Health.

[51] De Lepeleere S., De Bourdeaudhuij I., Van Stappen V., Huys N., Latomme J., Androutsos O., Manios Y., Cardon G., Verloigne M. (2018). Parenting practices as a mediator in the association between family socio-economic status and screen-time in primary schoolchildren: A Feel4Diabetes Study. Int. J. Environ. Res. Public Health.

[52] Cohen J. (1988). Statistical Power Analysis for the Behavioral Sciences.

[53] Chavakis A., Pagalou M., Rigoutsos S., Ventsanou F. Physical activity and educational practices of preschool teachers and physical activity of children in day-care centers. Proceedings of the 27th International Congress on Physical Education & Sport Science.

[54] Vanderloo L.M. (2014). Screen-viewing among preschoolers in childcare: A systematic review. BMC Pediatr..

[55] Kourlaba G., Kondaki K., Liarigkovinos T., Manios Y. (2009). Factors associated with television viewing time in toddlers and preschoolers in Greece: The GENESIS study. J. Public Health.

[56] (2001). American Academy of Pediatrics. Committee on Public Education. American Academy of Pediatrics: Children, adolescents, and television. Pediatrics.

[57] Economou C., Kaitelidou D., Kentikelenis A., Maresso A., Sissouras A. (2014). The Impact of the Financial Crisis on Health and the Health System in Greece.

[58] Vandoros S., Hessel P., Leone T., Avendano M. (2013). Have health trends worsened in Greece as a result of the financial crisis? A quasi-experimental approach. Eur. J. Public Health.

[59] Hellenic Confederation of Professionals, Craftsmen and Merchants (2014). Research on Household Income and Expenses.

[60] Organization for Economic Cooperation and Development (OECD) (2014). Society at A Glance 2014: OECD Social Indicators.

[61] Goranitis I., Siskou O., Liaropoulos L. (2014). Health policy making under information constraints: An evaluation of policy responses to the economic crisis in Greece. Health Policy.

[62] Du Boys C., Padovani E., Monti A. Vulnerability factors shaping municipal resilience throughout the global financial crisis: Comparing Italy and France. Proceedings of the EGPA Annual Conference.

[63] Venetsanou F., Kambas A. (2017). Physical activity promotion in Greek preschools: The gap between theory and practice. Early Child. Educ. J..

[64] Papatheodorou C., Papanastasiou S. (2017). The State of the Children in Greece Report 2017. The Children of the Crisis.

[65] Bevan A.L., Reilly S.M. (2011). Mothers’ efforts to promote healthy nutrition and physical activity for their preschool children. J. Pediatr. Nurs..

[66] Virto L.M. (2018). How do the Spanish families face to crisis? The types and consequences of coping strategies. METSZETEK.

[67] Sigurdsen P., Berger S., Heymann J. (2011). The Effects of Economic Crises on Families Caring for Children: Understanding and Reducing Long-term Consequences. Dev. Policy Rev..

[68] Joseph E.D., Kracht C.L., St Romain J., Allen A.T., Barbaree C., Martin C.K., Staiano A.E. (2019). Young Children’s Screen Time and Physical Activity: Perspectives of Parents and Early Care and Education Center Providers. Glob. Pediatr. Health.

[69] Kaur N., Gupta M., Malhi P., Grover S. (2019). Screen Time in Under-five Children. Indian Pediatr..

[70] Active Healthy Kids Canada (2014). Report on Physical Activity: Is Canada in the Running. http://dvqdas9jty7g6.cloudfront.net/reportcard2014/AHKC_2014_ReportCard_ENG.pdf.

[71] Downing K.L., Hinkley T., Salmon J., Hnatiuk J.A., Hesketh K.D. (2017). Do the correlates of screen time and sedentary time differ in preschool children?. BMC Public Health.

[72] Ji M., Tang A., Zhang Y., Zou J., Zhou G., Deng J., Yang L., Li M., Chen J., Qin H. (2018). The Relationship between Obesity, Sleep and Physical Activity in Chinese Preschool Children. Int. J. Environ. Res. Public Health.

[73] Veldhuis L., van Grieken A., Renders C.M., HiraSing R.A., Raat H. (2014). Parenting Style, the Home Environment, and Screen Time of 5-Year-Old Children; The ‘Be Active, Eat Right’ Study. PLoS ONE.

[74] Tandon P.S., Zhou C., Lozano P., Christakis D.A. (2011). Preschoolers’ total daily screen time at home and by type of child care. J. Pediatr..

[75] UNICEF Office of Research (2014). Children of the Recession: The Impact of the Economic Crisis on Child Well-Being in Rich Countries.

[76] Anand V., Downs S.M., Bauer N.S., Carroll A.E. (2014). Prevalence of Infant Television Viewing and Maternal Depression Symptoms. J. Dev. Behav. Pediatr..

[77] Emond J.A., Tantum L.K., Gilbert-Diamond D., Kim S.J., Lansigan R.K., Neelon S.B. (2018). Household chaos and screen media use among preschool-aged children: A cross-sectional study. BMC Public Health.

[78] Stephenson A., McDonough S.M., Murphy M.H., Nugent C.D., Mair J.L. (2017). Using computer, mobile and wearable technology enhanced interventions to reduce sedentary behaviour: A systematic review and meta-analysis. Int. J. Behav. Nutr. Phys. Act..

[79] Christakis D.A. (2009). The effects of infant media usage: What do we know and what should we learn. Acta Paediatr..

[80] Zimmerman F.J., Christakis D.A., Meltzoff A.N. (2007). Television and DVD/Video Viewing in Children Younger Than 2 Years. Arch. Pediatr. Adolesc. Med..

[81] Thompson D.A., Polk S., Cheah C.S., Vandewater E.A., Johnson S.L., Chrismer M.C., Tschann J.M. (2015). Maternal beliefs and parenting practices regarding their preschool child’s television viewing: An exploration in a sample of low-income Mexican-origin mothers. Clin. Pediatr. (Phila).

[82] Detnakarintra K., Trairatvorakul P., Pruksananonda C., Chonchaiya W. (2019). Positive mother-child interactions and parenting styles were associated with lower screen time in early childhood. Acta Paediatr..

[83] Lauricella A.R., Wartella E.A., Rideout V.J. (2015). Young children’s screen time: The complex role of parent and child factors. J. Appl. Dev. Psychol..

[84] Møller N.C., Christensen L.B., Mølgaard C., Ejlerskov K.T., Pfeiffer K.A., Michaelsen K.F. (2017). Descriptive analysis of preschool physical activity and sedentary behaviors—A cross sectional study of 3-year-olds nested in the SKOT cohort. BMC Public Health.

[85] Roscoe C.M.P., James R.S., Duncan M.J. (2019). Accelerometer-based physical activity levels differ between week and weekend days in British preschool children. J. Funct. Morphol. Kinesiol..

[86] Ott E., Vanderloo L.M., Tucker P. (2019). Physical activity and screen-viewing policies in Canadian childcare centers. BMC Public Health.

[87] Tremblay M.S., Chaput J.-P., Adamo K.B., Aubert S., Barnes J.D., Choquette L., Duggan M., Faulkner G., Goldfield G.S., Gray C.E. (2017). Canadian 24-hour movement guidelines for the early years (0–4 years): An integration of physical activity, sedentary behaviour, and sleep. BMC Public Health.

[88] Barbosa S. (2016). Physical activity of preschool children: A review. J. Physiother. Phys. Rehabil..

[89] Gerritsen S., Morton S.M.B., Wall C.R. (2016). Physical activity and screen use policy and practices in childcare: Results from a survey of early childhood education services in New Zealand. Australian and New Zealand. J. Public Health.

[90] Gregoriadis A., Tsigilis N., Grammatikopoulos V., Kouli O. (2016). Comparing quality of childcare and kindergarten centres: The need for a strong and equal partnership in the Greek early childhood education system. Early Child Dev. Care.

